# Imiquimod Reverses Chronic Toxoplasmosis-Associated Behavioral and Neurocognitive Anomalies in a Rat Model

**DOI:** 10.3390/biomedicines12061295

**Published:** 2024-06-11

**Authors:** Shaymaa Itani, Maguy Hamie, Reem El Jammal, Wassim Abdine, Mark Doumit, Adib Charafeddine, Marwan El-Sabban, Cindy Patinote, Carine Masquefa, Pierre-Antoine Bonnet, Makram Obeid, Hiba El Hajj

**Affiliations:** 1 Department of Experimental Pathology, Immunology and Microbiology, Faculty of Medicine, American University of Beirut, Beirut 1107 2020, Lebanon; ski02@mail.aub.edu (S.I.); mh242@aub.edu.lb (M.H.); wa88@aub.edu.lb (W.A.); 2 Department of Anatomy, Cell Biology and Physiological Sciences, Faculty of Medicine, American University of Beirut, Beirut 1107 2020, Lebanon; rme84@mail.aub.edu (R.E.J.); mnd09@mail.aub.edu (M.D.); me00@aub.edu.lb (M.E.-S.); makobeid@iu.edu (M.O.); 3 College of Pharmacy, American University of Iraq-Baghdad, Baghdad 10071, Iraq; adib.charafeddine@auib.edu.iq; 4 Institut des Biomolécules Max Mousseron (IBMM), UMR 5247, CNRS, ENSCM, Université de Montpellier, 34090 Montpellier, France; cindy.patinote@umontpellier.fr (C.P.); carine.masquefa@umontpellier.fr (C.M.); pierre-antoine.bonnet1@umontpellier.fr (P.-A.B.)

**Keywords:** cytokines, imiquimod, immune response toxoplasmosis, toxoplasmosis-associated behavioral disorders

## Abstract

*Toxoplasma gondii* is the etiologic agent of toxoplasmosis, a highly prevalent parasitosis. *Toxoplasma gondii* (*T. gondii*) transits in the brain from acute (AT) to chronic toxoplasmosis (CT), under host immune control. In immunocompromised patients, reactivation of CT is potentially life-threatening. Behavioral and neurological complications have been associated with CT. Furthermore, an effective treatment targeting CT is still lacking. We previously reported the efficacy of imiquimod against CT. Here, we demonstrate the molecular effects of imiquimod or imiquimod followed by the clinically used combination of sulfadiazine and pyrimethamine (SDZ + PYR) on CT-associated behavior in a rat model. Imiquimod decreased the number of cysts in the brains of chronically infected rats due to an induced reactivation of bradyzoites into tachyzoites. Importantly, this decrease was more pronounced in rats treated with imiquimod followed by SDZ + PYR. Rats chronically infected with *T. gondii* exhibited an anxiety-like behavior. Notably, treatment with imiquimod reversed this behavior aberrancy, with even a more pronounced effect with imiquimod followed by SDZ/PYR. Similarly, rats chronically infected with *T. gondii* exhibited learning deficits, and imiquimod alone or followed by SDZ/PYR reversed this behavior. Our results enhance our knowledge of the implications of CT on behavioral aberrancies and highlight the potency of imiquimod followed by SDZ + PYR on these CT-associated complications.

## 1. Introduction

*Toxoplasma gondii* (*T. gondii*) is an obligate intracellular parasite that infects a broad range of hosts, including approximately one-third of the worldwide human population [1]. The prevalence of this infection varies according to region [2] and can reach an alarming percentage of 80% in certain areas [1]. In the United States of America, the Center for Disease Control and Prevention reported that more than 40 million people are infected with *T. gondii* and classified toxoplasmosis among the neglected parasitic infections requiring public health action control [3].

*T. gondii* exhibits three distinct infectious stages: tachyzoites (responsible for acute toxoplasmosis (AT) leading to tissue damage), bradyzoites (responsible for chronic toxoplasmosis (CT) manifested as tissue cysts), and sporozoites (infective forms found in oocysts shed in cats’ feces). AT develops following tachyzoites’ spread and replication. Tachyzoites reach the brain and the skeletal muscles and convert into bradyzoite cysts, initiating CT, after the onset of the host immune system (reviewed in [4]). The brain harbors the highest cyst load in humans and murine models [5,6]. These intraneuronal cysts are controlled but not eliminated by the immune system (reviewed in [7,8]), even after long-term chemotherapeutic options [9]. In immunocompromised patients, reactivation of CT still occurs and can be fatal [8,10,11,12,13]. Hence, a balance between the host immune response and the parasitic modulators is at the core of the cysts’ persistence, control, and progression (reviewed in [8,14]).

Until recently, *T. gondii* persistence in the brains of healthy individuals was regarded as clinically asymptomatic, while the effects of CT are not fully explored. However, an increasing number of associations have been made between various medical conditions and *T. gondii* infections with only a little molecular proof. These comprise primary neurological complications, behavioral and psychiatric disorders, and some brain cancers [15,16,17,18,19,20,21,22,23,24]. In humans, an increasing body of literature indicates that CT is associated with aberrant host behavior [25] and that *T. gondii* seropositivity is allied with worse immediate and delayed verbal learning, language proficiency, executive functioning, processing speed, sustained attention, working memory, as well as global cognition in older adults [26]. Moreover, CT influences the progression of psychiatric disorders such as schizophrenia, bipolar disorder, and obsessive-compulsive disorder [24,27,28]. In *T. gondii*-infected rodents, behavioral peculiarities were also reported, where infected animals exhibited attenuated aversion and fear and did not flee a cat’s urine odor (reviewed in [29]). Furthermore, C57BL/6 mice infected with the type II strain of *T. gondii* exhibited hyperactivity, anxiety, and depressive-like behavior in both early and long-term CT, and these behavioral alterations were paralleled with an upregulation of several cytokines and chemokines [30].

Treatment of toxoplasmosis remains limited to general anti-parasitic/anti-bacterial drugs (reviewed [31,32,33,34]). The recommended first-line therapy remains the synergistic combination of pyrimethamine (PYR), an inhibitor of dihydrofolate reductase enzyme, and sulfadiazine (SDZ), an inhibitor of the dihydropteroate synthase [33,35,36]. SDZ/PYR reversed behavioral and neurocognitive changes and resolved locomotor alterations, anxiety, and depressive-like behavior. Furthermore, this combination partially or transiently ameliorated hyperactivity and habituation memory loss in rodent models [37]. Yet, this combination is mostly active on AT, with no or little effect on CT. Anti-*Toxoplasma* drugs and compounds identified numerous target-based drug screens [38]. Nevertheless, most of these drugs were effective only against tachyzoites, and only very few target bradyzoites [38]. Hence, to date, there is no approved therapy that eliminates tissue cysts responsible for CT [38,39,40].

Imiquimod is an FDA-approved immune-modulatory drug for topical use against some viral infections [41]. Imiquimod is an immune response activator [42,43] that binds TLR-7/TLR-8 [44,45,46,47], activating the innate immune response by induction, synthesis, and the release of pro-inflammatory cytokines from monocytes and other immune cells [42,45]. These include IFN-α, IL-6, and TNF-α [48]. Importantly, imiquimod proved efficient against parasitic infections such as cutaneous leishmaniasis [41,45,49,50,51] and toxoplasmosis [33,52]. We indeed previously demonstrated that imiquimod holds a high potency against AT and CT in murine models [52]. Treatment with imiquimod during AT reduced the number of brain cysts while rendering the remaining ones un-infectious. Importantly, treatment with imiquimod, post-establishment of CT, significantly reduced the number of brain cysts, leading to a delay or abortion of reactivation [52]. At the molecular level, imiquimod upregulated the expression of TLRs and the activation of the MyD88 pathway, resulting in the induction of the immune response to control reactivation [52].

We used a rat model with chronic toxoplasmosis and tested the effect of imiquimod on CT and its associated behavioral changes using the open field test to study the anxiety-like behavior and the Morris water maze test to assess learning deficits. Moreover, we examined the effect of imiquimod followed by the combination of SDZ/PYR on the reactivation of CT and the reversal of CT-associated behavioral disorders.

## 2. Materials and Methods

### 2.1. Preparation of Drugs

Imiquimod (2.5 mg/kg) (SIGMA I5159-200MG; Livonia, MI, USA) was dissolved in dimethylsulfoxide (DMSO) and freshly diluted in an equal volume of lipofundin-MCT/LCT 20% emulsion (B. Braun Melsungen, AG/D-34209; Melsungen, Germany) prior to the rats’ intraperitoneal injection. Sulfadiazine (200 mg/L) (SIGMA S8626-100G; Roedermark, Germany) was dissolved in water and administered in the rats’ drinking water. Pyrimethamine (10 mg/kg) [9] (SIGMA 46706–250 mg; Roedermark, Germany) was dissolved in ethanol and freshly diluted in an equal volume of lipofundin-MCT/LCT 20% emulsion before its intraperitoneal administration to rats.

### 2.2. In Vitro Maintenance of Parasites in Human Foreskin Fibroblasts

The 76K strain *Toxoplasma gondii* was kindly provided by Dr. Mathieux Gissot. Tachyzoites were serially passaged in Human Foreskin Fibroblasts (HFFs) (American Type Culture Collection (ATCC, Manassas, MA, USA)—CRL 1634) cultured in Dulbecco’s Modified Eagle’s Medium (DMEM) (SIGMA; Roedermark, Germany), supplemented with 10% Fetal Bovine Serum (FBS), 1% penicillin-streptomycin, and 1% glutamine (SIGMA; Roedermark, Germany).

### 2.3. Protein Gel Electrophoresis and Western Blotting

A sample of freshly collected tachyzoites of the 76K strain was boiled in Laemmli SDS PAGE sample buffer and separated on 12% polyacrylamide gels. Proteins were transferred to nitrocellulose membranes (BIO-RAD Cat# 162-0112; Hercules, CA, USA) at 30 V overnight using a BIO-RAD transfer unit. Nitrocellulose membranes were cut into strips that were saturated/blocked for 1 h in 5% non-fat dry milk in wash buffer (15 mM Tris-HCl (pH 8), 150 mM NaCl, and 0.05% Tween 20). An average of 100 μL of blood withdrawn from rats was centrifuged at 13,000 rpm for 15 min. Then, strips were incubated with sera from different rats (10 μL in 1 mL of 5% non-fat milk in wash buffer) at 4 °C to check for seropositivity. After washing, the strips were then incubated with Goat Anti-Rat secondary antibody IgG, peroxidase-conjugated (Invitrogen stock concentration 0.8 mg/mL, dilution of 1:500; Waltham, MA, USA), and revealed with luminol-based chemiluminescent substrate (BIO-RAD Cat# 170-5061; Hercules, CA, USA), which binds to the secondary antibody and produces light detected by autoradiography.

### 2.4. Immunofluorescence Assay

For immunofluorescence assay, Human Foreskin Fibroblast (HFF) cells infected with the 76K strain (1:3 parasite to cell ratio) for 24 h were fixed with 4% paraformaldehyde in PBS for 20 min. Permeabilization of cells was then performed in 0.2% Triton for 10 min. Following one PBS wash, the blocking of cells with 10% FBS in PBS for 30 min was performed. Harvested serum from the infected rats was used as a primary antibody (dilution 1:10) against tachyzoites. Goat Anti-Rat secondary antibody IgG (Alexa Fluor 488 2 mg/mL Abcam ab 150157, dilution 1:500; Cambridge, UK) was used to stain the tachyzoites (green). Staining of nuclei (blue) was performed using 1 µg/mL of Hoechst 33,342 trihydrochloride trihydrate solution (Invitrogen, H33342; Waltham, MA, USA) for 5 min. Coverslips were then mounted onto slides using a Prolong anti-fade kit (Invitrogen, P36930; Waltham, MA, USA). Images were acquired with confocal microscopy using the confocal microscope (Zeiss LSM 710, Göttingen, Germany), and the analysis of the images was performed using Zeiss Zen software (Zen 2.3 Sp1).

### 2.5. Transcriptional Expression Analysis

**Figure 1:**
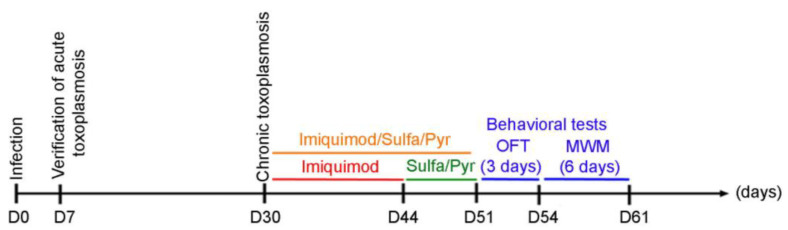
Timeline schedule for the assessment of the effect of imiquimod followed by the combination of sulfadiazine and pyrimethamine and imiquimod on chronic toxoplasmosis-associated behavioral disorders. Briefly, on Day 0, Sprague Dawley rats were injected with 3 million tachyzoites/rat of 76K. On Day 7, the acute phase of the infection was verified, and the rats were allowed to reach chronic toxoplasmosis (Day 30). A group of rats then were treated with imiquimod alone every other day for 2 weeks, the second group was treated for 1 week with the combination of Sulfadiazine and Pyrimethamine, and the third group was treated with imiquimod every other day for 2 weeks followed by 1-week treatment with the combination of sulfadiazine and pyrimethamine. After that, behavioral tests were performed with an open field test (OFT) for 3 days, followed by the Morris water maze (MWM) for 6 days.

Quantitative Real-Time PCR (qRT PCR) was performed using a CFX 384 machine (BIO-RAD; Hercules, CA, USA). Different primers were used to detect transcripts in the brains of chronically infected rats with *T. gondii* upon treatment with imiquimod, sulfadiazine/pyrimethamine, or imiquimod followed by sulfadiazine/pyrimethamine (Timeline Figure 1). In qRT-PCR, individual reactions were prepared with 150 ng of cDNA, 0.25 µM of each of the respective forward and reverse primers (Table 1, Macrogen, Seoul, Republic of Korea), and SYBR Green PCR Master Mix to a final volume of 10 µL. The PCR reaction consisted of a DNA denaturation step for 3 min at 95 °C, followed by 40 cycles (denaturation at 95 °C for 15 s, annealing at 57 °C for 60 s, extension at 72 °C for 30 s). Glyceraldehyde-3-Phosphate dehydrogenase (GAPDH) was used as a housekeeping gene. Reactions were performed in duplicates, and the expression of individual genes was normalized to GAPDH threshold cycle (Ct) values. The threshold cycle (Ct) corresponds to the cycle at which there is a significant detectable increase in fluorescence. Data were plotted by calculating ΔCt (Ct_target gene_ − Ct_GAPDH_). The percentage of expression was calculated according to the Livak method: 2^−ΔΔCt^ [53].

### 2.6. Ethic Statement

This animal study was reviewed and approved by the Institutional Animal Care and Utilization Committee (IACUC) of the American University of Beirut (AUB) (Permit Number: #: 21-10-590). All rats were housed in a specific pathogen-free facility with a 12 h ON/OFF light cycle. Humane endpoints were fully respected as per AUB IACUC following the Association for Assessment and Accreditation of Laboratory Animal Care International guidelines and guide of animal care use book (Guide, NRC 2011).

### 2.7. Animals and Experimental Design

One-month-old male Sprague Dawley rats were used in our study (Timeline Figure 1). On day 0, rats were intraperitoneally injected with 3 million tachyzoites of the 76K type II strain of *T. gondii*. On day 7, blood samples were collected from the periorbital sinus of each rat to verify the establishment of AT. The seropositivity of rats against *T. gondii* was assessed using immunofluorescence and western blot assays. CT in seropositive rats was allowed to develop for 4 weeks [54]. Afterward, the imiquimod treatment was administered intraperitoneally at the dose of 2.5 mg/kg/day every other day [52] for 2 weeks. One group of rats received imiquimod at a dose of 2.5 mg/kg/day every other day for 2 weeks, followed by the combination of SDZ/PYR for 1 week, while another control group received the combination of SDZ/PYR for one week. For the behavioral testing, rats were divided into 6 groups: uninfected controls untreated (UCU), uninfected controls treated with imiquimod (UCT), untreated rats infected with *T. gondii* (TU), rats infected with *T. gondii* and treated with imiquimod (TT), infected rats treated with the combination of SDZ/PYR (TC), and infected rats treated with imiquimod followed by the combination of SDZ/PYR (TTC). Behavioral analysis was started on day 46 with 2 consecutive behavioral tests: an open-field test (OFT for 3 days) and a Morris water maze test (MWM for 6 days) (Timeline Figure 1). On day 61, rats were sacrificed, their brains were collected, and bradyzoite cysts were quantified using an optimized Percoll method [55,56]. Molecular analysis of the brain immune response in respective rat groups was performed.

### 2.8. Behavioral Tests

#### 2.8.1. Open Field Test (OFT)

The open field test was performed to monitor the locomotor activity, anxiety, and exploratory-like behaviors. Accordingly, the test was conducted for 3 consecutive days, with a 5-min session each day. Rats were placed in an opaque plexiglass square field (W: 80 cm, L: 80 cm, H: 40 cm). The ceiling light was turned off and a circular neon lamp was placed above the center of the field to maximize contrast between the center and the periphery. On day 1 of the testing, one small object (cube) was placed in the center of the field’s floor, and then a novel object (ball, then bottle) was introduced on each of the two remaining days. On the day of the test, each rat was placed in the nearest corner to the most recently added novel object, and its movement was recorded. The walls and floor surfaces of the apparatus were cleaned with odorless detergent and then a 70% alcohol solution between each trial. The rats’ movements (distance traveled, time spent in each zone (peripheral versus central), time spent exploring central objects) were separately measured and analyzed using the SMART video tracking 3.0 software (Panlab, Harvard Apparatus, Holliston, MA, USA) [57,58].

#### 2.8.2. Morris Water Maze (MWM)

The Morris water maze test was used to assess hippocampal-dependent visuospatial navigation. The test was performed over consecutive days, with day 1 being the habituation day, followed by days of spatial acquisition testing, a probe trial, and a visible platform on the final day. The setup consisted of a blue circular plastic pool, 150 cm in diameter and 80 cm in height (Coulbourn Instruments, Holliston, MA, USA), that was filled with water (25 °C) to a depth of 30 cm. The pool was divided into 4 quadrants (Northeast, Northwest, Southeast, Southwest) and surrounded by visual cues adhered to the room’s walls. On the day of habituation, rats were allowed to swim freely for 2 min. During the spatial acquisition trials for 5 days, an “invisible platform” (transparent plexiglass cylinder) was placed 2 cm below the water surface in the Northeast quadrant. Each rat was placed in water and allowed to swim for 2 min to reach the platform. In case the rat did not find the platform, it was placed on it for 30 s. A total of 4 daily trials were performed for every rat, each with a 30-s rest time in between, and four equidistant immersion landmarks from the platform with their sequence changed each day. On day 6, the probe trial was performed to assess the retention of the spatial learning, where each rat was allowed to swim freely for 2 min without the platform and then was immersed in the quadrant opposite the quadrant that previously had the platform. On the same day, the rats were allowed to swim to a visible platform (gray opaque plastic cylinder) placed in the Southeast quadrant, with 4 attempts per rat and 4 different immersion positions in the Northwest quadrant, to assess for potential motor or visual dysfunction that may affect the experiment. At the end of the daily trials, each rat was placed under a heating lamp to dry, and the pool was periodically cleaned. Every trial was video recorded and analyzed using the SMART video tracking 3.0 software (Panlab, Harvard Apparatus, Holliston, MA, USA) to measure the escape latency period from immersion in the pool until reaching the platform and the time spent in each quadrant [57,58].

### 2.9. Statistics

Statistical analysis was performed using the Graph Pad Prism Software (Version 8.4.3) (*t*-test, one-way ANOVA with post hoc Fisher’s Least Significant Difference, two-way ANOVA with post hoc Fisher’s Least Significant Difference). *, **, ***, **** indicate *p*-values ≤ 0.05, 0.01, 0.001, and 0.0001, respectively. *p*-values less than 0.05 were considered significant. 

## 3. Results

### 3.1. Establishment of Chronic Toxoplasmosis in Sprague Dawley Rat Model

Rats were injected intraperitoneally with a dose of 3 million tachyzoites. While serum from not-infected rats showed no reactivity (Figure S1A, upper panel), sera from all tested infected rats reacted with in vitro precultured tachyzoites with immunofluorescence assay (Figure S1A, lower panel). Similarly, a complex profile of bands, with a major band at 30 kDa that corresponded to the surface antigen-1 (SAG-1), was detected using sera from infected rats with the 76K strain, as compared to sera from non-infected rats. A representative western blot of sera from 7 rats infected with *T. gondii* is shown in (Figure S1B). Collectively, our results demonstrate that Sprague Dawley rats successfully developed AT following infection with *T. gondii* tachyzoites.

### 3.2. Imiquimod Reduces the Number of Brain Cysts, Induces the Conversion of Bradyzoites to Tachyzoites, and Stimulates an Inflammatory Immune Response in Chronically Infected Rats

**Figure 2:**
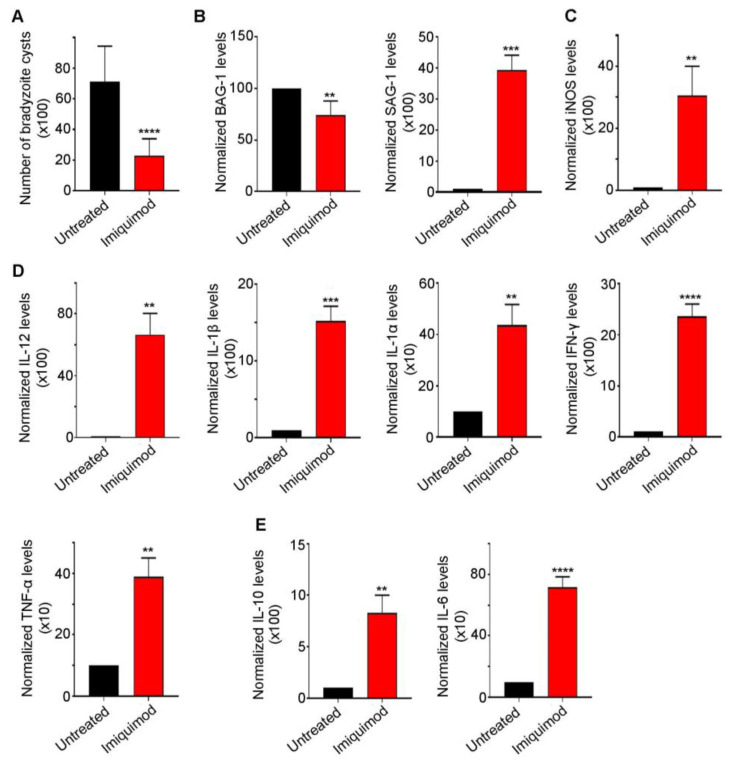
Imiquimod exhibits a potent effect against chronic toxoplasmosis. (**A**) Number of bradyzoite cysts in the brain of untreated rats (black) and rats treated with imiquimod (red). Each rat was infected with 3 million tachyzoites of the 76K strain and treated with imiquimod with a dose of 2.5 mg/kg/day. A *t*-test was performed to validate the significance. **** indicate *p*-values ≤ 0.0001. *p*-values less than 0.05 were considered significant. SD (±) is reported. The results depict the sum of 4 independent experiments. (Untreated, *n* = 24 brains; imiquimod, *n* = 26 brains). (**B**) Quantitative Real-Time PCR of BAG-1 (**left** panel) and SAG-1 (**right** panel) from total brain extracts of untreated rats (black) and rats treated with imiquimod (red). BAG-1 and SAG-1 expression was normalized to GAPDH. *t*-test was performed to validate significance. **, *** indicate *p*-values ≤ 0.01 and 0.001, respectively. *p*-values of less than 0.05 were considered significant. SD (±) is reported. The results depict one representative experiment among two independent ones. (Untreated, *n* = 8 brains; imiquimod, *n* = 8 brains). (**C**) Quantitative Real-Time PCR for i-NOS from total brain extracts of untreated rats (black) and rats treated with imiquimod (red). i-NOS expression was normalized to GAPDH. *t*-test was performed to validate significance. ** indicate *p*-values ≤ 0.01, respectively. *p*-values of less than 0.05 were considered significant. SD (±) is reported. The results depict one representative experiment among two independent ones. (Untreated, *n* = 6 brains; Imiquimod, *n* = 6 brains). (**D**) Quantitative Real-Time PCR of IL-12, IL-1β, IL-1α, IFN-γ, and TNF-α from the brains of untreated rats (black) and rats treated with imiquimod (red). IL-12, IL-1β, IL-1α, IFN-γ, and TNF-α expression was normalized to GAPDH. The results depict one representative experiment among two independent ones. (Untreated, *n* = 6 brains; imiquimod, *n* = 6 brains). (**E**) Quantitative Real-Time PCR of IL-10 and IL-6 from the brains of untreated rats (black) and rats treated with imiquimod (red). IL-10 and IL-6 expression was normalized to GAPDH. *t*-test was performed to validate the significance. **, ***, **** indicate *p*-values ≤ 0.01, 0.001, and 0.0001, respectively. *p*-values less than 0.05 were considered significant. SD (±) is reported. The results depict one representative experiment among two independent ones. (Untreated, *n* = 6 brains; imiquimod, *n* = 6 brains).

We previously reported that post-establishment of CT, imiquimod significantly abridged the number of brain cysts in mice. This effect was due to reactivation resulting in the induction of the immune response to control reactivated *Toxoplasma* foci [52]. Similarly, in this study, we demonstrated a sharp decrease in brain cyst number upon treatment with imiquimod (Figure 2A). This decrease was due to a conversion from bradyzoites into tachyzoites, as manifested by the significant decrease in Bradyzoite antigen-1 (BAG-1) transcript levels (*p*-value < 0.01) and the sharp increase in the tachyzoite surface antigen SAG-1 (*p*-value < 0.001) (Figure 2B). Imiquimod-induced reactivation was concurrent with a significant increase in i-NOS (*p*-value < 0.01) (Figure 2C) and the pro-inflammatory cytokines IL-12 (*p*-value < 0.01), IL-1β (*p*-value < 0.001), IL-1α (*p*-value < 0.01), IFN-γ (*p*-value < 0.0001), and TNF-α (*p*-value < 0.01) (Figure 2D). A concomitant increase in the anti-inflammatory response was also noted (Figure 2E). Indeed, imiquimod induced a significant increase in IL-10 (*p*-value < 0.01) and IL-6 (*p*-value < 0.0001) transcript levels (Figure 2E), presumably to lessen the pro-inflammatory response induced by reactivated foci. Altogether, the obtained results in a rat model of CT were exactly similar to those obtained in mice upon treatment with imiquimod.

### 3.3. Treatment with Imiquimod Followed by the Combination of Sulfadiazine and Pyrimethamine Is More Effective Than Imiquimod Alone on CT

**Figure 3:**
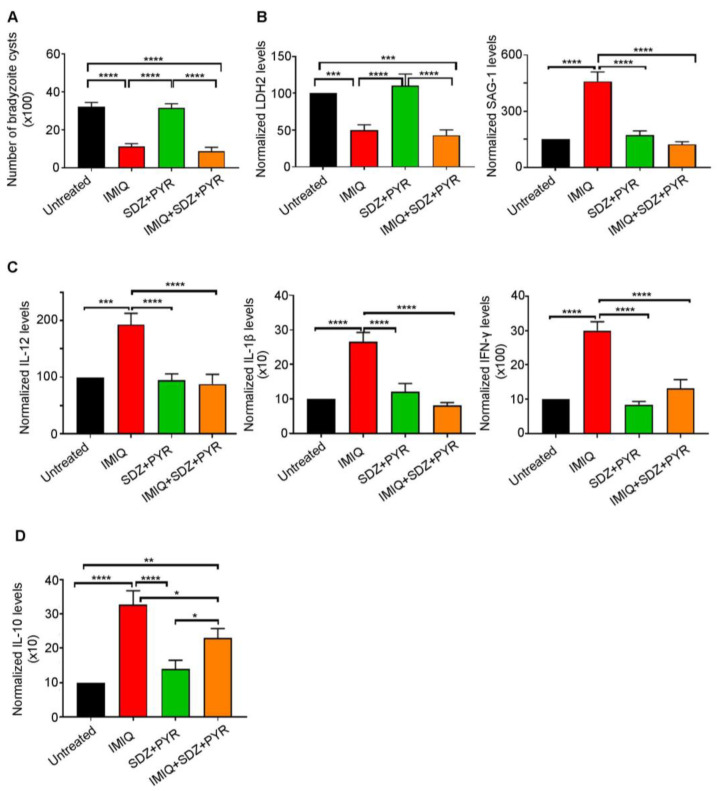
Imiquimod followed by the combination of sulfadiazine and pyrimethamine exhibits a more potent effect against chronic toxoplasmosis than with imiquimod alone. (**A**) Number of bradyzoite cysts in the brains of untreated rats (black), rats treated with imiquimod (red), rats treated with the combination of sulfadiazine and pyrimethamine (green), and rats treated with the combination of imiquimod, sulfadiazine, and pyrimethamine (orange). Each rat was infected with 3 million tachyzoites of the 76K strain. Afterward, imiquimod treatment was administered intraperitoneally at the dose of 2.5 mg/kg/day every other day for 2 weeks to a group of rats. Another group of rats received imiquimod at a dose of 2.5 mg/kg/day every other day for 2 weeks followed by the combination of SDZ/PYR for 1 week, while another control group received the combination of SDZ/PYR for one week. Ordinary one-way repeated measures ANOVA with post hoc Fisher’s least significant difference (LSD) was used to validate significance. **** indicate *p*-values ≤ 0.0001, respectively. *p*-values less than 0.05 were considered significant. SEM (±) is reported. The results depict the sum of 2 independent experiments. (Untreated, *n* = 10 brains; IMIQ, *n* = 10 brains; SDZ + PYR, *n* = 10 brains; IMIQ + SDZ + PYR, *n* = 10 brains). (**B**) Quantitative Real-Time PCR of LDH2 (**left** panel) and SAG-1 (**right** panel) from total brain extracts of untreated rats (black), rats treated with imiquimod (red), rats treated with the combination of sulfadiazine and pyrimethamine (green), and rats treated with the combination of imiquimod, sulfadiazine and pyrimethamine (orange). LDH-2 and SAG-1 expression was normalized to GAPDH. Ordinary one-way repeated measures ANOVA with post hoc Fisher’s (LSD) was used to validate significance. ***, **** indicate *p*-values ≤ 0.001 and 0.0001, respectively. *p*-values less than 0.05 were considered significant. SEM (±) is reported. The results depict the sum of 2 independent experiments. For Quantitative Real-Time PCR of LDH2 (Untreated, *n* = 9 brains; IMIQ, *n* = 11 brains; SDZ + PYR, *n* = 10 brains; IMIQ + SDZ + PYR, *n* = 11 brains), for Quantitative Real-Time PCR of SAG-1 (Untreated, *n* = 9 brains; IMIQ, *n* = 11 brains; SDZ + PYR, *n* = 10 brains; IMIQ + SDZ + PYR, *n* = 11 brains). (**C**) Quantitative Real-Time PCR of IL-12, IL-1β and IFN-γ from the total brain extracts of untreated rats (black), rats treated with imiquimod (red), rats treated with the combination of sulfadiazine and pyrimethamine (green), and rats treated with the combination of imiquimod, sulfadiazine, and pyrimethamine (orange). IL-12, IL-1β, and IFN-γ expression was normalized to GAPDH. Ordinary one-way repeated measures ANOVA with post hoc Fisher’s (LSD) was used to validate significance. ***, **** indicate *p*-values ≤ 0.001 and 0.0001, respectively. *p*-values less than 0.05 were considered significant. SEM (±) is reported. The results depict the sum of 2 independent experiments. For Quantitative Real-Time PCR of IL-12 (Untreated, *n* = 10 brains; IMIQ, *n* = 10 brains; SDZ + PYR, *n* = 11 brains; IMIQ + SDZ + PYR, *n* = 15 brains), for Quantitative Real-Time PCR of IL-1β (Untreated, *n* = 12 brains; IMIQ, *n* = 10 brains; SDZ + PYR, *n* = 11 brains; IMIQ + SDZ + PYR, *n* = 15 brains), for Quantitative Real-Time PCR of IFN-γ (Untreated, *n* = 11 brains; IMIQ, *n* = 11 brains; SDZ + PYR, *n* = 9 brains; IMIQ + SDZ + PYR, *n* = 16 brains). (**D**) Quantitative Real-Time PCR of IL-10 from the total brain extracts of untreated rats (black), rats treated with imiquimod (red), rats treated with the combination of sulfadiazine and pyrimethamine (green), and rats treated with the combination of imiquimod, sulfadiazine, and pyrimethamine (orange). IL-10 expression was normalized to GAPDH. Ordinary one-way repeated measures ANOVA with post hoc Fisher’s least significant difference (LSD) was used to validate significance. *, **, **** indicate *p*-values ≤ 0.05, 0.01, and 0.0001, respectively. *p*-values less than 0.05 were considered significant. SEM (±) is reported. The results depict the sum of 2 independent experiments. (Untreated, *n* = 12 brains; IMIQ, *n* = 11 brains; SDZ + PYR, *n* = 12 brains; IMIQ + SDZ + PYR, *n* = 13 brains).

The recommended first-line therapy of toxoplasmosis is the synergistic combination of SDZ/PYR [33,35,36]. This combination is mostly active on AT, with no or little effect on CT [38,39,40]. Imiquimod-induced reactivation of bradyzoites to tachyzoites prompted us to examine the efficacy of adding SDZ/PYR after treatment with imiquimod (Timeline Figure 1). While SDZ/PYR did not exhibit any effect on the brain cyst number (3168 cysts as compared to 3210 cysts in the untreated group), imiquimod followed by SDZ/PYR drastically and significantly decreased the number of cysts to 882 cysts as compared to 3210 cysts in untreated groups (*p*-value < 0.0001) and 1116 cysts in rats treated with imiquimod alone (Figure 3A, Table 2). Remarkably, treatment with SDZ/PYR after imiquimod, but not this combination alone, sharply diminished the transcript levels of SAG-1 in the brains of infected rats, as compared to rats treated with imiquimod alone (*p*-value < 0.0001) (Figure 3B, Table 2). In addition, the transcription levels of the bradyzoite-expressed marker LDH-2 [59] significantly decreased in the brains of chronically infected rats after treatment with imiquimod or imiquimod followed by SDZ/PYR (*p*-value < 0.001), while the combination SDZ/PYR did not exhibit any significant change in the transcripts of this bradyzoite marker (Figure 3B). Importantly, treatment with imiquimod followed by SDZ/PYR significantly reversed the imiquimod-induced immune response. Indeed, transcript levels of the pro-inflammatory cytokines IL-12, IL-1β, and IFN-γ significantly decreased in the brains of chronically infected rats treated with imiquimod followed by SDZ/PYR, as compared to those treated with imiquimod alone (*p*-value < 0.0001) (Figure 3C). Similarly, a significant decrease in the transcript levels of the anti-inflammatory cytokine IL-10 was denoted in the brains of the group of rats treated with imiquimod followed by SDZ/PYR, as compared to the group of rats treated with imiquimod alone (*p*-value < 0.05) (Figure 3D). Altogether, our results reinforce all data showing that SDZ/PYR is not effective on CT and clearly demonstrate the higher potency of imiquimod followed by SDZ/PYR on CT when compared to imiquimod alone.

### 3.4. Anxiety-like Behavior Exhibited by Chronically Infected Rats Is Reversed by Imiquimod and Further Enhanced When Followed by the Combination of Sulfadiazine and Pyrimethamine

**Figure 4:**
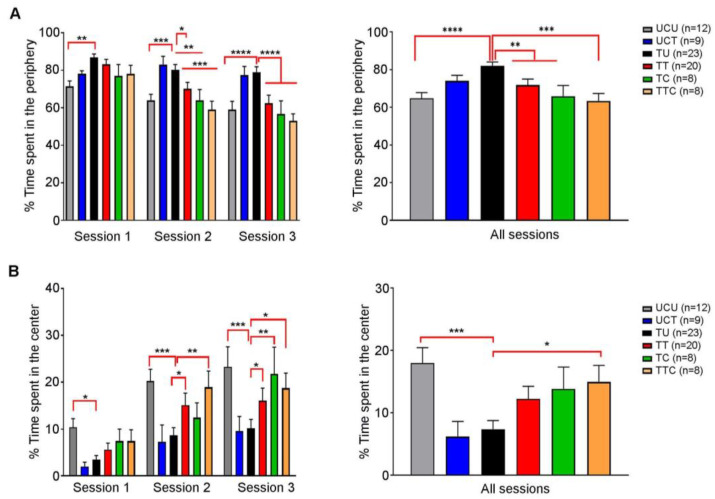
Rats chronically infected with *T. gondii* exhibit an anxiety-like behavior, and imiquimod reverses this behavior with a better effect when followed by the combination of sulfadiazine and pyrimethamine. (**A**) Open field test. On the left side, in session 1, the percentage of time spent in the periphery was higher in the TU group compared to the UCU group, in addition to significance in other groups (UCU.UCT **, UCU.TT *). In session 2, the percentage of time spent in the periphery was higher in the TU group compared to the UCU group and the treated groups, in addition to the significance in other groups (UCU.UCT **, UCT.TT *, UCT.TC **, UCT.TTC ***). In session 3, the percentage of time spent in the periphery was higher in the TU group compared to the UCU group and the treated groups in addition to the significance in other groups (UCU.UCT **, UCT.TT **, UCT.TC **, UCT.TTC ***). On the right side, in all sessions, the percentage of time spent in the periphery was higher in the TU group compared to the UCU group and the treated groups, in addition to the significance in other groups (UCU.UCT ***, UCT.TT *, UCT.TC **, UCT.TTC ***). (**B**) Open field test. On the left side, in session 1, the percentage of time spent in the center was higher in the UCU group compared to the TU group, in addition to significance in other groups (UCU.UCT *). In session 2, the percentage of time spent in the center was higher in the UCU, TTC, and TT groups compared to the TU group, in addition to the significance in other groups (UCU.UCT **, UCT.TT *, UCT.TTC *). In session 3, the percentage of time spent in the center was higher in the UCU, TTC, and TT groups compared to the TU group, in addition to the significance in other groups (UCU.UCT **, UCU.TT * UCT.TC **, UCT.TTC *). On the right side, in all sessions, the percentage of time spent in the center was higher in the UCU and TTC groups compared to the TU group in addition to the significance in other groups (UCU.UCT **, UCT.TTC *). *, **, ***, **** indicate *p*-values ≤ 0.05, 0.01, 0.001, and 0.0001, respectively. For the **left** panels, two-way repeated measures ANOVA with post hoc Fisher’s (LSD) was used to validate significance. *p*-values less than 0.05 were considered significant. SEM ± are reported. For the **right** panels, ordinary one-way repeated measures ANOVA with post hoc Fisher’s least significant difference (LSD) was used to validate significance. (UCU, *n* = 12; UCT, *n* = 9; TU, *n* = 23; TT, *n* = 20, TC, *n* = 8, TTC, *n* = 8).

Behavioral changes were reported in *T. gondii*-infected rodents (reviewed in [29,30]. It was also demonstrated that SDZ/PYR reversed behavioral and neurocognitive changes and resolved locomotor alterations, anxiety, and depressive-like behavior in rodent models [37]. We investigated the effect of imiquimod or imiquimod followed by SDZ/PYR on the exploratory and anxiety-like behaviors of chronically infected rats with *T. gondii* using the OFT test (Timeline presented in Figure 1). This test is based on the conflict that arises between the rats’ natural innate aversion to open-lit areas and their drive to explore. In all sessions, there was no significant difference in the total distance traveled by the different groups of rats throughout the whole OFT (Figure S2). Over the three sessions, the untreated *Toxoplasma* group (TU) spent significantly more time in the periphery as compared to the uninfected control (UCU) (*p*-value < 0.01, *p*-value < 0.001, and *p*-value < 0.0001, respectively) (Figure 4A left panel; Table 3). During the first session, no significant difference was denoted in the time the treated groups spent in the periphery, as compared to the TU group (ns) (Figure 4A left panel). However, during the second session, both the TT and TTC groups spent less time in the periphery as compared to the TU group (*p*-value < 0.05, and *p*-value < 0.001, respectively) (Figure 4B, left panel). Similar results were obtained over the third session (*p*-value < 0.0001 for both groups) (Figure 4B, left panel). In all the sessions, the TU group spent significantly more time in the periphery as compared to the UCU group (*p*-value < 0.0001) (Figure 4A, right panel), indicating that the *Toxoplasma*-infected rats exhibit an anxiety-like behavior. In all sessions, the TT and the TTC groups spent less time in the periphery compared to the TU group (*p*-value < 0.01) and with more statistical significance in the TTC group (*p*-value < 0.001) (Figure 4A, right panel; Table 3), indicating that imiquimod treatment reverses the anxiety-like behavior induced by *Toxoplasma* infection, with a better effect for imiquimod treatment followed by the SDZ/PYR. Moreover, over the three sessions, the TU group spent significantly less time in the center, as compared to the UCU group (*p*-value < 0.05, *p*-value < 0.001, and *p*-value < 0.001, respectively) (Figure 4B, left panel). While in the first session, the treatment groups were comparable to TU, in subsequent sessions they spent more time in the center compared to the TU group; in both the second session (*p*-value < 0.05 and *p*-value < 0.01, respectively) and the third session (*p*-value < 0.05 for both groups) for TT and TTC and in the third session for TC (*p*-value < 0.01 for both groups) (Figure 4B, left panel). Cumulatively over the 3 sessions, the TU group spent significantly less time in the center as compared to UCU (*p*-value < 0.001) and TTC, which were comparable (Figure 4B, right panel). These results indicate a *Toxoplasma*-induced decrease in exploratory behaviors that is reversed by the treatments.

Collectively, our results demonstrate that *T. gondii* chronic infection induces anxiety-like behavior in rats, and imiquimod treatment reverses this behavior with a better effect for imiquimod treatment followed by the combination of SDZ/PYR.

### 3.5. Learning Deficits in Chronically Infected Rats Are Reversed upon Treatment with Imiquimod Followed by the Combination of Sulfadiazine and Pyrimethamine

**Figure 5:**
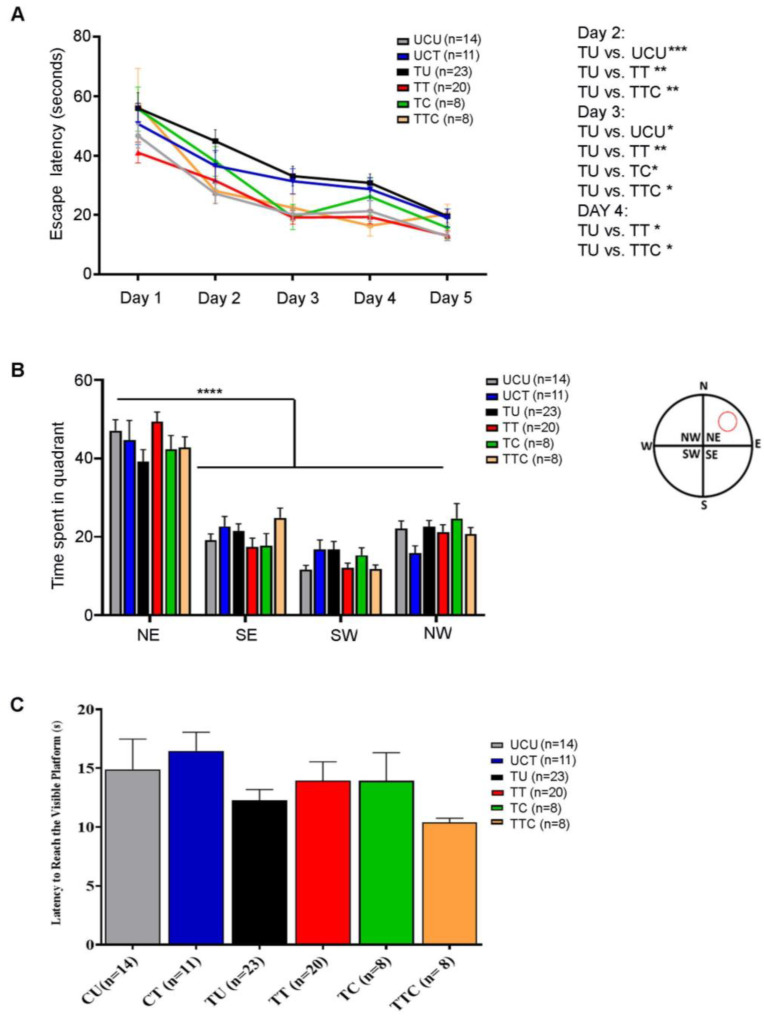
Rats chronically infected with *T. gondii* exhibit learning deficits in the Morris water maze, and imiquimod alone or followed by the combination of sulfadiazine and pyrimethamine reverses this behavior. (**A**) Latency to reach the escape platform during 5 training days. The TU group took more time to reach the invisible platform (higher escape latency) compared to the UCU group, especially on days 2 and 3. The TT and TTC groups had a similar performance to the UCU group in learning the place of the invisible platform during the 5-day spatial acquisition training (**B**). In the probe trial test, the 6 groups showed similar preference for the Northeast Quadrant. The red circle in the water maze diagram corresponds to the previous location of the platform (N: North, E: East, S: South, W: West, NE: Northeast, NW: Northwest, SE: Southeast, SW: Southwest). (**C**) Visual acuity assessment. All the groups had comparable latency in reaching the visible platform, indicating comparable visual and motor functions in all groups. For (**A**,**B**), two-way repeated measures ANOVA with post hoc Fisher’s (LSD) was used to validate significance. For (**C**), ordinary one-way repeated measures ANOVA with post hoc Fisher’s least significant difference (LSD) was used to validate significance. *, **, ***, **** indicate *p*-values ≤ 0.05, 0.01, 0.001, and 0.0001, respectively. *p*-values less than 0.05 were considered significant. SEM ± are reported. (UCU, *n* = 14; UCT, *n* = 11; TU, *n* = 23; TT, *n* = 20, TC, *n* = 8, TTC, *n* = 8).

The Morris water maze test was performed to evaluate visuospatial navigation and learning. During the five training days (Timeline described in Figure 1), all the rats gradually learned to reach the escape platform. The TU group required more time to reach the invisible platform (higher escape latency) as compared to the UCU group, and this trend reached statistical significance on days 2 (*p*-value < 0.001) and 3 (*p*-value < 0.05) (Figure 5A). The TT and TTC groups were faster in learning the place of the escape platform than the TU group with (*p*-value < 0.01) for both groups on day 2, (*p*-value < 0.01 for the TT group and *p*-value < 0.05 for the TTC group) on day 3, and (*p*-value < 0.05) for both groups on day 4 (Figure 5A, Table 4). The probe trial was performed to check the retention of learning. In the probe trial subtest, all groups showed a similar preference for the Northeast quadrant where the platform was previously located, indicating an intact retention of place learning (*p*-value < 0.0001) (Figure 5B, Table 4). In the visible platform subtest, the escape latencies were comparable between all the groups without any detectable visual or locomotor impairment (Figure 5C, Table 4). Altogether, these results show that *T. gondii* chronic infection induces visuospatial learning deficits in rats, and imiquimod treatment in addition to imiquimod treatment followed by the combination of SDZ/PYR reverses this behavioral disturbance.

## 4. Discussion

Several associations between CT and behavioral neurological complications were reported (reviewed in [16]). However, these associations were mostly based on epidemiological studies correlating seropositivity against *T. gondii* and the respective behavioral disorders (reviewed in [16]).

Despite the high prevalence of CT worldwide, the current therapies against toxoplasmosis remain limited to general anti-parasitic/anti-bacterial drugs (reviewed in [31,32,33,34]) and mainly target AT. Indeed, there is no clinically effective drug against CT or its associated neurological and behavioral complications, and only a few drugs target the bradyzoites in vitro or in preclinical models (reviewed in [33]). An ideal drug against toxoplasmosis should not only be effective against the proliferative tachyzoite stage of the parasite but also against the tissue cyst stage, especially as CT is the most prevalent form of this infection. An ideal drug can also modulate the host immune function, which plays a key role in CT maintenance and reactivation. We previously established the potency of the immunomodulatory drug imiquimod against murine CT and offered a molecular understanding of this efficacy [52]. Since rats are well-established animal models in behavioral studies [60], we reproduced a rat model of CT and demonstrated the potency of imiquimod alone or followed by the combination of SDZ/PYR on CT-associated behavioral aberrations. Indeed, imiquimod significantly decreased the number of cysts in the brains of CT-infected rats, and this effect was further enhanced upon treatment with imiquimod followed by the combination of SDZ/PYR. Similar to the obtained results in mice [52], this decrease in cyst number was due to the imiquimod-induced reactivation of bradyzoites into tachyzoites and was concurrent with an upregulation of the inducible nitric oxide synthase and the proinflammatory cytokines (IL-12, IL-1α and IL-1β, TNF-α and IFN-γ). These results in the rat model of CT are in line with the previous results reporting imiquimod-induced upregulation of several proinflammatory cytokines to control the reactivated foci [52]. This result is also in line with the well-established role of imiquimod in activating the innate immune response with induction, synthesis, and the release of pro-inflammatory cytokines (IFN-α, IL-6, and TNF-α) [48] from monocytes and other immune cells [42,45] and with the published literature reporting the role of proinflammatory cytokines in mounting a protective immune response against *T. gondii* [7,61,62,63,64]. In that sense, IL-12 and IL-1β play a crucial role in the recruitment of natural killer cells and neutrophils that start producing IFN-γ until the recruitment of T, which produces the highest amount of this cytokine to combat the infection (reviewed in [63,64,65,66]). The upregulation of IFN-γ in the brains of imiquimod-treated rats presumably indicates the recruitment of both innate and adaptive immune cells to fight the reactivated foci. Moreover, it was previously reported that treatment of murine-established CT with imiquimod, followed by immunosuppression using dexamethasone, resulted in significant and prolonged survival despite the reactivation [52]. Importantly, imiquimod followed by SDZ/PYR attenuated the imiquimod-induced inflammatory state in the brains of CT-infected rats.

It is well-documented that CT is associated with neurological complications and behavioral and psychiatric disorders [15,16,17,18,19,20,21]. Human CT is associated with aberrant host behavior [25] and influences the progression of psychiatric disorders [27,28], partly due to altered dopamine levels, leading to imbalances related to mood control, sleep patterns, and even attention deficit disorder [67]. Using a panel of behavioral tests including OFT to study anxiety-like, hyperactivity-like, and exploratory-like behaviors and the Morris water maze (MWM) to assess hippocampal-dependent visuospatial navigation, we showed that *T. gondii* infection induces anxiety-like behavior in chronically infected rats. This was reflected by the greater time the infected rats spent in the periphery and the less time they spent exploring the center objects. This result was consistent with a study conducted on C57BL/6J mice chronically infected with tachyzoite of *Toxoplasma* Prugniaud strain, by which mice exhibited an increased anxiety phenotype using OFT [68]. Indeed, these C57BL/6 mice exhibited hyperactivity, anxiety-like, and depressive-like behaviors in both early and long-term chronic toxoplasmosis infection, and these behavioral alterations were paralleled with an upregulation of several cytokines and chemokines [30]. In contrast to a study conducted on AT by infecting Wistar rats with the type I *T. gondii* RH strain where no significant difference in anxiety-like behavior was reported between the controls and *T. gondii* infected rats [60], our study demonstrated the effect of CT on inducing anxiety-like behavior.

Importantly, we demonstrate that imiquimod reverses the anxiety-like behavior induced by *Toxoplasma* infection and that imiquimod treatment followed by the SDZ/PYR exhibited a more prominent effect on this aberrant behavior. This result is in line with the documented effect of SDZ/PYR on reversing behavioral and neurocognitive changes and resolving locomotor alterations, anxiety, and depressive-like behavior in rodent models [37]. Finally, using the Morris water maze to evaluate visuospatial navigation and learning, we demonstrated that untreated chronically infected rats with *T. gondii* exhibited a higher escape latency and took more time to reach the invisible platform, which is indicative of a learning deficit. Imiquimod treatment alone or followed by the combination of SDZ/PYR reversed this behavior. This result was contradictory to a study conducted on rats with latent toxoplasmosis in which rats exhibited alterations to memory and learning; however, no significant difference in search latencies to find the hidden platform between the control group and the *T. gondii*-infected group was detected [69].

In conclusion, our results enhance our knowledge of the implications of chronic toxoplasmosis on behavioral aberrancies and highlight the potency of imiquimod followed by SDZ/PYR on these chronic toxoplasmosis-associated neurological complications.

## Figures and Tables

**Table 1:** Summary of primers used in RT-PCR.

Primer		Sequence
Rat GAPDH	Forward	5′-GGCAAGTTCAACGGCACAG-3′
	Reverse	5′-CGCCAGTAGACTCCACGAC-3′
SAG-1	Forward	5′-ACTCACCCAACAGGCAAATC-3′
	Reverse	5′-GAGACTAGCAGAATCCCCCG-3′
BAG-1	Forward	5 ′-GCGGAGAAAGTGGACGATGATGG-3′
	Reverse	5′-GTCGGGCTTGTAATTACTCGGG-3′
Rat IL-1α	Forward	5′-AAGACAAGCCTGTGTTGCTGAAGG-3′
	Reverse	5′-TCCCAGAAGAAAATGAGGTCGGTC-3′
Rat IL-1β	Forward	5′-CACCTCTCAAGCAGAGCACAG-3′
	Reverse	5′-GGGTTCCATGGTGAAGTCAAC-3′
Rat IL-6	Forward	5′-TCCTACCCCAACTTCCAATGCTC-3′
	Reverse	5′-TTGGATGGTCTTGGTCCTTAGCC-3′
Rat TNF-α	Forward	5′-AAATGGGCTCCCTCTCATCAGTTC-3′
	Reverse	5′-TCTGCTTGGTGGTTTGCTACGAC-3′
Rat IL-10	Forward	5′-GAAAAATTGAACCACCCGGCA-3′
	Reverse	5′-TTCCAAGGAGTTGCTCCCGT-3′
Rat INF-γ	Forward	5′-CAAGGCACACTCATTGAAAGC-3′
	Reverse	5′-TACTCGTAGCGGTTCAAGCTC-3′
Rat IL-12	Forward	5′-TGGAGTCATAGGCTCTGGA-3′
	Reverse	5′-GTCGTGGTCGAAGAAGTAG-3′
Rat i-NOS	Forward	5′-GCTTGCCCCTGGAACTTT-3′
	Reverse	5′-CGAACGGGGACCTTCAAA-3′
LDH-2	Forward	5′-ACAATGGCCCAGGCATTCT-3′
	Reverse	5′-CAATAAACATATCGTGAAGCCCATA-3′

**Table 2:** Summary of number of cysts and reactivation status per tested condition.

Condition	Number of Rats	Number of Cysts	Reactivation
Untreated	12	3210	No
Imiquimod	11	1116	Yes
Sulfadiazine + Pyrimethamine	12	3168	No
Imquimod followed by Sulfadiazine + Pyrimethamine	13	882	No

**Table 3:** Summary of obtained results on CT-associated anxiety-like behavior per group of tested rats.

Condition	CT-Associated Anxiety-like Behavior	
	Number of Rats	Effect
Uninfected Untreated (UCU)	12	No anxiety-like behavior
Uninfected treated with imiquimod (UCT)	9	Moderate effect on anxiety-like behavior
Infected untreated (TU)	23	Strong effect on anxiety-like behavior
Infected treated with imiquimod (TT)	20	Reversed anxiety-like behavior
Infected treated with sulfadiazine/pyrimethamine (TC)	8	Reversed anxiety-like behavior
Infected treated with imiquimod followed by sulfadiazine/ pyrimethamine (TTC)	8	Best reversed anxiety-like behavior

**Table 4:** Summary of obtained results on CT-associated learning deficits per group of tested rats.

Condition	CT-Associated Learning Deficits	
	Number of Rats	Effect
Uninfected Untreated (UCU)	14	No learning deficit
Uninfected treated with imiquimod (UCT)	11	Slight learning deficit
Infected untreated (TU)	23	Strong learning deficits
Infected treated with imiquimod (TT)	20	No learning deficits
Infected treated with sulfadiazine/pyrimethamine (TC)	8	No learning deficits
Infected treated with imiquimod followed by sulfadiazine/pyrimethamine (TTC)	8	No learning deficits

## Data Availability

The original contributions presented in this study are included in the article/Supplementary Material; further inquiries can be directed to the corresponding author.

## Supplementary Materials

The following supporting information can be downloaded at: https://www.mdpi.com/article/10.3390/biomedicines12061295/s1, Figure S1: (A,B) Sprague Dawley rats successfully developed acute toxoplasmosis. (A) Immunofluorescence Assay to test the immune reactivity of infected rats on pre-cultured tachyzoites in HFF using rat serum as a primary antibody. Goat Anti-Rat secondary antibody IgG (Alexa Fluor 488, 2 mg/mL, Abcam ab 150157, dilution 1:500) was used to stain the tachyzoites (Green) (first column). Nuclei of cells were stained with DAPI (Blue). Transmission photomultiplier tube (TPMT) contrast phase, and merged figures are also presented (columns 2 and 3). The result shown depict one representa-tive experiment among 2 independent ones. (B) Western Blot Assay to test the immune reactivity of infected rats on tachyzoite extracts, strip 1 corresponds to the serum of uninfected rat and strips 2 to 8 corresponds to sera from prototypes of infected rats. All rats included in this study were tested for seropositivity. Any seronegative rat was discarded from the study; Figure S2: (A,B) Total distance travelled by Open Field Test. In all sessions, no significant difference in the total distance travelled by the different group of rats in the whole OFT. One-way repeated measures ANOVA with post hoc Fisher (LSD) was used to validate significance. *, **, ***, **** indicate *p* values ≤ 0.05; 0.01 and 0.001, 0.0001, respectively. *p*-values less than 0.05 were considered significant. SEM ± are reported. For both panels, ordinary one-way repeated measures ANOVA with post hoc Fisher least (LSD) was used to validate significance. For (A), UCU, *n* = 10; UCT, *n* = 9; TU, *n* = 22; TT, *n* = 20. For (B), UCU, *n* = 6; TU, *n* = 8; TT, *n* = 6; TC, *n* = 8, TTC, *n* = 8.

## References

[1] Robert-Gangneux F., Dardé M.-L. (2012). Epidemiology of and Diagnostic Strategies for Toxoplasmosis. Clin. Microbiol. Rev..

[2] Calero-Bernal R., Gennari S.M., Cano S., Salas-Fajardo M.Y., Ríos A., Álvarez-García G., Ortega-Mora L.M. (2023). Anti-*Toxoplasma gondii* Antibodies in European Residents: A Systematic Review and Meta-Analysis of Studies Published between 2000 and 2020. Pathogens.

[3] Ben-Harari R.R., Connolly M.P. (2019). High burden and low awareness of toxoplasmosis in the United States. Postgrad. Med..

[4] Schlüter D., Barragan A. (2019). Advances and Challenges in Understanding Cerebral Toxoplasmosis. Front. Immunol..

[5] Fabiani S., Pinto B., Bonuccelli U., Bruschi F. (2015). Neurobiological studies on the relationship between toxoplasmosis and neuropsychiatric diseases. J. Neurol. Sci..

[6] Konradt C., Ueno N., Christian D.A., Delong J.H., Pritchard G.H., Herz J., Bzik D.J., Koshy A.A., McGavern D.B., Lodoen M.B. (2016). Endothelial cells are a replicative niche for entry of *Toxoplasma gondii* to the central nervous system. Nat. Microbiol..

[7] Blanchard N., Dunay I.R., Schluter D. (2015). Persistence of *Toxoplasma gondii* in the central nervous system: A fine-tuned balance between the parasite, the brain and the immune system. Parasite Immunol..

[8] Matta S.K., Rinkenberger N., Dunay I.R., Sibley L.D. (2021). *Toxoplasma gondii* infection and its implications within the central nervous system. Nat. Rev. Microbiol..

[9] Neville A.J., Zach S.J., Wang X., Larson J.J., Judge A.K., Davis L.A., Vennerstrom J.L., Davis P.H. (2015). Clinically Available Medicines Demonstrating Anti-*Toxoplasma* Activity. Antimicrob. Agents Chemother..

[10] Bannoura S., El Hajj R., Khalifeh I., El Hajj H. (2018). Acute disseminated encephalomyelitis and reactivation of cerebral toxoplasmosis in a child: Case report. IDCases.

[11] Kodym P., Malý M., Beran O., Jilich D., Rozsypal H., Machala L., Holub M. (2015). Incidence, immunological and clinical characteristics of reactivation of latent *Toxoplasma gondii* infection in HIV-infected patients. Epidemiol. Infect..

[12] Montoya J.G., Liesenfeld O. (2004). Toxoplasmosis. Lancet.

[13] Basavaraju A. (2016). Toxoplasmosis in HIV infection: An overview. Trop. Parasitol..

[14] Frickel E.M., Hunter C.A. (2021). Lessons from Toxoplasma: Host responses that mediate parasite control and the microbial effectors that subvert them. J. Exp. Med..

[15] Ngô H.M., Zhou Y., Lorenzi H., Wang K., Kim T.-K., Zhou Y., El Bissati K., Mui E., Fraczek L., Rajagopala S.V. (2017). *Toxoplasma* Modulates Signature Pathways of Human Epilepsy, Neurodegeneration & Cancer. Sci. Rep..

[16] Daher D., Shaghlil A., Sobh E., Hamie M., Hassan M.E., Moumneh M.B., Itani S., El Hajj R., Tawk L., El Sabban M. (2021). Comprehensive Overview of *Toxoplasma gondii*-Induced and Associated Diseases. Pathogens.

[17] Martinez V.O., de Mendonça Lima F.W., De Carvalho C.F., Menezes-Filho J.A. (2018). *Toxoplasma gondii* infection and behavioral outcomes in humans: A systematic review. Parasitol. Res..

[18] Virus M.A., Ehrhorn E.G., Lui L.M., Davis P.H. (2021). Neurological and Neurobehavioral Disorders Associated with *Toxoplasma gondii* Infection in Humans. J. Parasitol. Res..

[19] Bisetegn H., Debash H., Ebrahim H., Mahmood N., Gedefie A., Tilahun M., Alemayehu E., Mohammed O., Feleke D.G. (2023). Global seroprevalence of *Toxoplasma gondii* infection among patients with mental and neurological disorders: A systematic review and meta-analysis. Health Sci. Rep..

[20] Gaskell E.A., Smith J.E., Pinney J.W., Westhead D.R., McConkey G.A. (2009). A unique dual activity amino acid hydroxylase in *Toxoplasma gondii*. PLoS ONE.

[21] Webster J.P., McConkey G.A. (2010). *Toxoplasma gondii*-altered host behaviour: Clues as to mechanism of action. Folia Parasitol.

[22] Johnson H.J., Koshy A.A. (2020). Latent Toxoplasmosis Effects on Rodents and Humans: How Much is Real and How Much is Media Hype?. mBio.

[23] Johnson S.K., Johnson P.T.J. (2021). Toxoplasmosis: Recent Advances in Understanding the Link between Infection and Host Behavior. Annu. Rev. Anim. Biosci..

[24] Rosado D., Intriago B., Loor E., Alcívar F., Avila J., Sotomayor M., Villacres L., Faytong-Haro M. (2024). Associations between *Toxoplasma gondii* seropositivity and psychopathological manifestations in schizophrenic patients: A single-center study from Ecuador. PLoS ONE.

[25] Milne G., Webster J.P., Walker M. (2020). *Toxoplasma gondii*: Underestimated Threat?. Trends Parasitol..

[26] Song G., Zhao Q., Chen H., Li M., Zhang Z., Qu Z., Yang C., Lin X., Ma W., Standlee C.R. (2024). *Toxoplasma gondii* seropositivity and cognitive functioning in older adults: An analysis of cross-sectional data of the National Health and Nutrition Examination Survey 2011–2014. BMJ Open.

[27] Severance E.G., Xiao J., Jones-Brando L., Sabunciyan S., Li Y., Pletnikov M., Prandovszky E., Yolken R. (2016). *Toxoplasma gondii*—A Gastrointestinal Pathogen Associated with Human Brain Diseases. Int. Rev. Neurobiol..

[28] Sutterland A.L., Fond G., Kuin A., Koeter M.W.J., Lutter R., van Gool T., Yolken R., Szoke A., Leboyer M., de Haan L. (2015). Beyond the association. Toxoplasma gondii in schizophrenia, bipolar disorder, and addiction: Systematic review and meta-analysis. Acta Psychiatr. Scand..

[29] Tong W.H., Pavey C., O’Handley R., Vyas A. (2021). Behavioral biology of *Toxoplasma gondii* infection. Parasit. Vectors.

[30] Castano Barrios L., Pinheiro A.P.D.S., Gibaldi D., Silva A.A., e Silva P.M.R., Roffê E., Santiago H.d.C., Gazzinelli R.T., Mineo J.R., Silva N.M. (2021). Behavioral alterations in long-term *Toxoplasma gondii* infection of C57BL/6 mice are associated with neuroinflammation and disruption of the blood brain barrier. PLoS ONE.

[31] Dunay I.R., Gajurel K., Dhakal R., Liesenfeld O., Montoya J.G. (2018). Treatment of Toxoplasmosis: Historical Perspective, Animal Models, and Current Clinical Practice. Clin. Microbiol. Rev..

[32] Konstantinovic N., Guegan H., Stäjner T., Belaz S., Robert-Gangneux F. (2019). Treatment of toxoplasmosis: Current options and future perspectives. Food Waterborne Parasitol..

[33] Hajj R.E., Tawk L., Itani S., Hamie M., Ezzeddine J., El Sabban M., El Hajj H. (2021). Toxoplasmosis: Current and Emerging Parasite Druggable Targets. Microorganisms.

[34] Rodriguez J.B., Szajnman S.H. (2023). An updated review of chemical compounds with anti-*Toxoplasma gondii* activity. Eur. J. Med. Chem..

[35] Lapinskas P.J., Ben-Harari R.R. (2019). Perspective on current and emerging drugs in the treatment of acute and chronic toxoplasmosis. Postgrad. Med..

[36] Remington J.S., Thulliez P., Montoya J.G. (2004). Recent Developments for Diagnosis of Toxoplasmosis. J. Clin. Microbiol..

[37] Castaño B.L., Silva A.A., Hernandez-Velasco L.L., Pinheiro A.P.D.S., Gibaldi D., Mineo J.R., Silva N.M., Lannes-Vieira J. (2022). Sulfadiazine Plus Pyrimethamine Therapy Reversed Multiple Behavioral and Neurocognitive Changes in Long-Term Chronic Toxoplasmosis by Reducing Brain Cyst Load and Inflammation-Related Alterations. Front. Immunol..

[38] Montazeri M., Sharif M., Sarvi S., Mehrzadi S., Ahmadpour E., Daryani A. (2017). A Systematic Review of In Vitro and In Vivo Activities of Anti-*Toxoplasma* Drugs and Compounds (2006–2016). Front. Microbiol..

[39] Alday P.H., Doggett J.S. (2017). Drugs in development for toxoplasmosis: Advances, challenges, and current status. Drug Des. Dev. Ther..

[40] Montazeri M., Mehrzadi S., Sharif M., Sarvi S., Tanzifi A., Aghayan S.A., Daryani A. (2018). Drug Resistance in *Toxoplasma gondii*. Front. Microbiol..

[41] Miller R.L., Gerster J., Owens M., Slade H., Tomai M. (1999). Review Article Imiquimod applied topically: A novel immune response modifier and new class of drug. Int. J. Immunopharmacol..

[42] Sauder D.N. (2000). Immunomodulatory and pharmacologic properties of imiquimod. J. Am. Acad. Dermatol..

[43] Suzuki H., Wang B., Shivji G.M., Toto P., Amerio P., Sauder D.N., Tomai M.A., Miller R.L. (2000). Imiquimod, a topical immune response modifier, induces migration of Langerhans cells. J. Investig. Dermatol..

[44] Hwang H., Min H., Kim D., Yu S.W., Jung S.J., Choi S.Y., Lee S.J. (2014). Imiquimod induces a Toll-like receptor 7-independent increase in intracellular calcium via IP_3_ receptor activation. Biochem. Biophys. Res. Commun..

[45] Raman V.S., Duthie M.S., Fox C.B., Matlashewski G., Reed S.G. (2012). Adjuvants for *Leishmania* vaccines: From models to clinical application. Front. Immunol..

[46] Smith K.J., Hamza S., Skelton H. (2003). The imidazoquinolines and their place in the therapy of cutaneous disease. Expert Opin. Pharmacother..

[47] Zhang W.W., Matlashewski G. (2008). Immunization with a Toll-like receptor 7 and/or 8 agonist vaccine adjuvant increases protective immunity against Leishmania major in BALB/c mice. Infect. Immun..

[48] Arevalo I., Ward B., Miller R., Meng T.C., Najar E., Alvarez E., Matlashewski G., Alejandro L.C. (2001). Successful treatment of drug-resistant cutaneous leishmaniasis in humans by use of imiquimod, an immunomodulator. Clin. Infect. Dis..

[49] Miranda-Verastegui C., Tulliano G., Gyorkos T.W., Calderon W., Rahme E., Ward B., Cruz M., Llanos-Cuentas A., Matlashewski G. (2009). First-line therapy for human cutaneous leishmaniasis in Peru using the TLR7 agonist imiquimod in combination with pentavalent antimony. PLoS Negl. Trop. Dis..

[50] El Hajj R., Youness H.B., Lachaud L., Bastien P., Masquefa C., Bonnet P.-A., El Hajj H., Khalifeh I. (2018). EAPB0503: An Imiquimod analog with potent in vitro activity against cutaneous leishmaniasis caused by Leishmania major and Leishmania tropica. PLoS Negl. Trop. Dis..

[51] Miranda-Verastegui C., Llanos-Cuentas A., Arevalo I., Ward B.J., Matlashewski G. (2005). Randomized, double-blind clinical trial of topical imiquimod 5% with parenteral meglumine antimoniate in the treatment of cutaneous leishmaniasis in Peru. Clin. Infect. Dis..

[52] Hamie M., Najm R., Deleuze-Masquefa C., Bonnet P.A., Dubremetz J.F., El Sabban M., El Hajj H. (2021). Imiquimod Targets Toxoplasmosis through Modulating Host Toll-like Receptor-MyD88 Signaling. Front. Immunol..

[53] Schmittgen T.D., Livak K.J. (2008). Analyzing real-time PCR data by the comparative C_T_ method. Nat. Protoc..

[54] Chew W.K., Wah M.J., Ambu S., Segarra I. (2012). *Toxoplasma gondii*: Determination of the onset of chronic infection in mice and the in vitro reactivation of brain cysts. Exp. Parasitol..

[55] Fritz H.M., Bowyer P.W., Bogyo M., Conrad P.A., Boothroyd J.C. (2012). Proteomic analysis of fractionated *Toxoplasma oocysts* reveals clues to their environmental resistance. PLoS ONE.

[56] Watts E.A., Dhara A., Sinai A.P. (2017). Purification *Toxoplasma gondii* Tissue Cysts Using Percoll Gradients. Curr. Protoc. Microbiol..

[57] Salah H., Medlej Y., Karnib N., Darwish N., Asdikian R., Wehbe S., Makki G., Obeid M. (2019). Methods in Emotional Behavioral Testing in Immature Epilepsy Rodent Models. Methods Mol. Biol..

[58] Medlej Y., Salah H., Wadi L., Saad S., Asdikian R., Karnib N., Ghazal D., Bashir B., Allam J., Obeid M. (2019). Overview on Emotional Behavioral Testing in Rodent Models of Pediatric Epilepsy. Methods Mol. Biol..

[59] Abdelbaset A.E., Fox B.A., Karram M.H., Ellah M.R.A., Bzik D.J., Igarashi M. (2017). Lactate dehydrogenase in *Toxoplasma gondii* controls virulence, bradyzoite differentiation, and chronic infection. PLoS ONE.

[60] Parvin Z., Iraj M.D., Minoo S., Fatemeh K. (2016). Effects of *Toxoplasma gondii* infection on anxiety, depression and ghrelin level in male rats. J. Parasit. Dis..

[61] Ihara F., Yamamoto M. (2024). The role of IFN-gamma-mediated host immune responses in monitoring and the elimination of *Toxoplasma gondii* infection. Int. Immunol..

[62] Melo M.B., Kasperkovitz P., Cerny A., Könen-Waisman S., Kurt-Jones E.A., Lien E., Beutler B., Howard J.C., Golenbock D.T., Gazzinelli R.T. (2010). UNC93B1 mediates host resistance to infection with *Toxoplasma gondii*. PLoS Pathog..

[63] Sasai M., Yamamoto M. (2019). Innate, adaptive, and cell-autonomous immunity against *Toxoplasma gondii* infection. Exp. Mol. Med..

[64] Sasai M., Yamamoto M. (2022). Anti-*Toxoplasma* host defense systems and the parasitic counterdefense mechanisms. Parasitol. Int..

[65] Yarovinsky F. (2008). Toll-like receptors and their role in host resistance to *Toxoplasma gondii*. Immunol. Lett..

[66] Sturge C.R., Yarovinsky F. (2014). Complex immune cell interplay in the gamma interferon response during *Toxoplasma gondii* infection. Infect. Immun..

[67] Xiao J., Li Y., Prandovszky E., Kannan G., Viscidi R.P., Pletnikov M.V., Yolken R.H. (2016). Behavioral Abnormalities in a Mouse Model of Chronic Toxoplasmosis Are Associated with MAG1 Antibody Levels and Cyst Burden. PLoS Negl. Trop. Dis..

[68] Tyebji S., Seizova S., Garnham A.L., Hannan A.J., Tonkin C.J. (2019). Impaired social behaviour and molecular mediators of associated neural circuits during chronic *Toxoplasma gondii* infection in female mice. Brain Behav. Immun..

[69] Daniels B.P., Sestito S.R., Rouse S.T. (2015). An expanded task battery in the Morris water maze reveals effects of *Toxoplasma gondii* infection on learning and memory in rats. Parasitol. Int..

