# Supplementary material for: Imiquimod Reverses Chronic Toxoplasmosis-Associated Behavioral and Neurocognitive Anomalies in a Rat Model
Source: Biomedicines. 2024 Jun 11;12(6):1295. doi: 10.3390/biomedicines12061295 (PMC11202296; doi:10.3390/biomedicines12061295)
Supplement: Supplementary file 1 [file biomedicines-12-01295-s001.zip › biomedicines-3027197-supplementary.pdf]

Supplementary Materials:

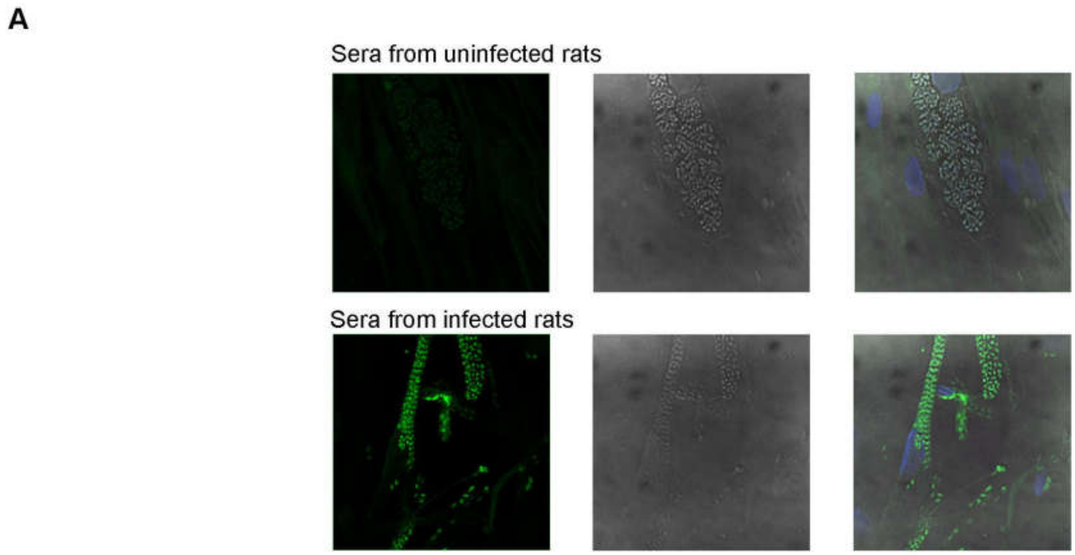

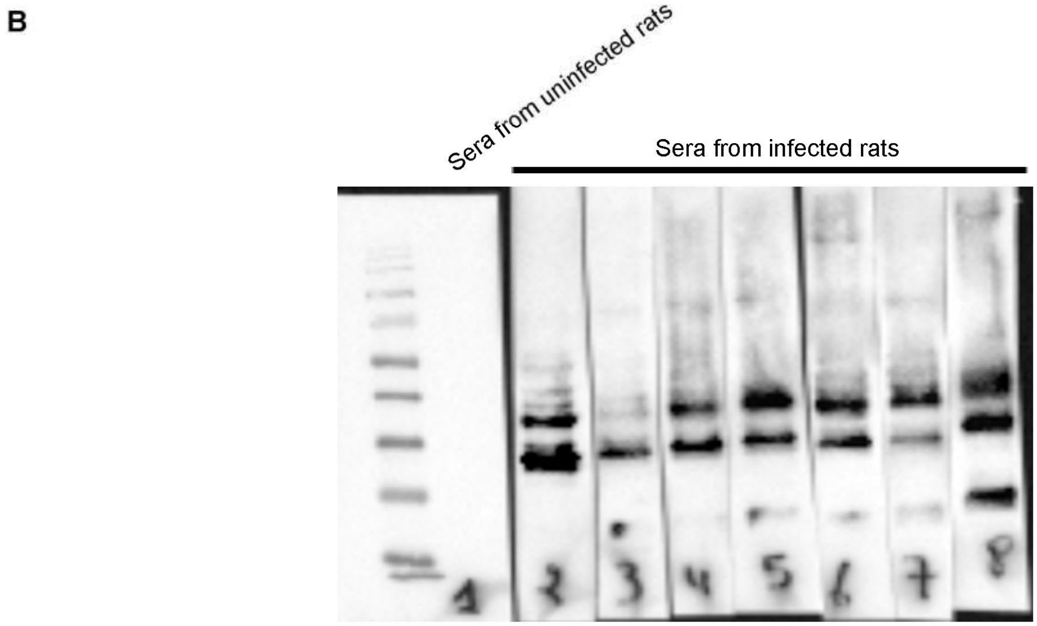

Figure S1. (**A**,**B**) Sprague Dawley rats successfully developed acute toxoplasmosis. (**A**) Immunofluorescence Assay to test the immune reactivity of infected rats on pre-cultured tachyzoites in HFF using rat serum as a primary antibody. Goat Anti-Rat secondary antibody IgG (Alexa Fluor 488, 2 mg/mL, Abcam ab 150157, dilution 1:500) was used to stain the tachyzoites (Green) (first column). Nuclei of cells were stained with DAPI (Blue). Transmission photomultiplier tube (TPMT) contrast phase, and merged figures are also presented (columns 2 and 3). The result shown depict one representative experiment among 2 independent ones. (**B**) Western Blot Assay to test the immune reactivity of infected rats on tachyzoite extracts, strip 1 corresponds to the serum of uninfected rat and strips 2 to 8 corresponds to sera from prototypes of infected rats. All rats included in this study were tested for seropositivity. Any seronegative rat was discarded from the study.

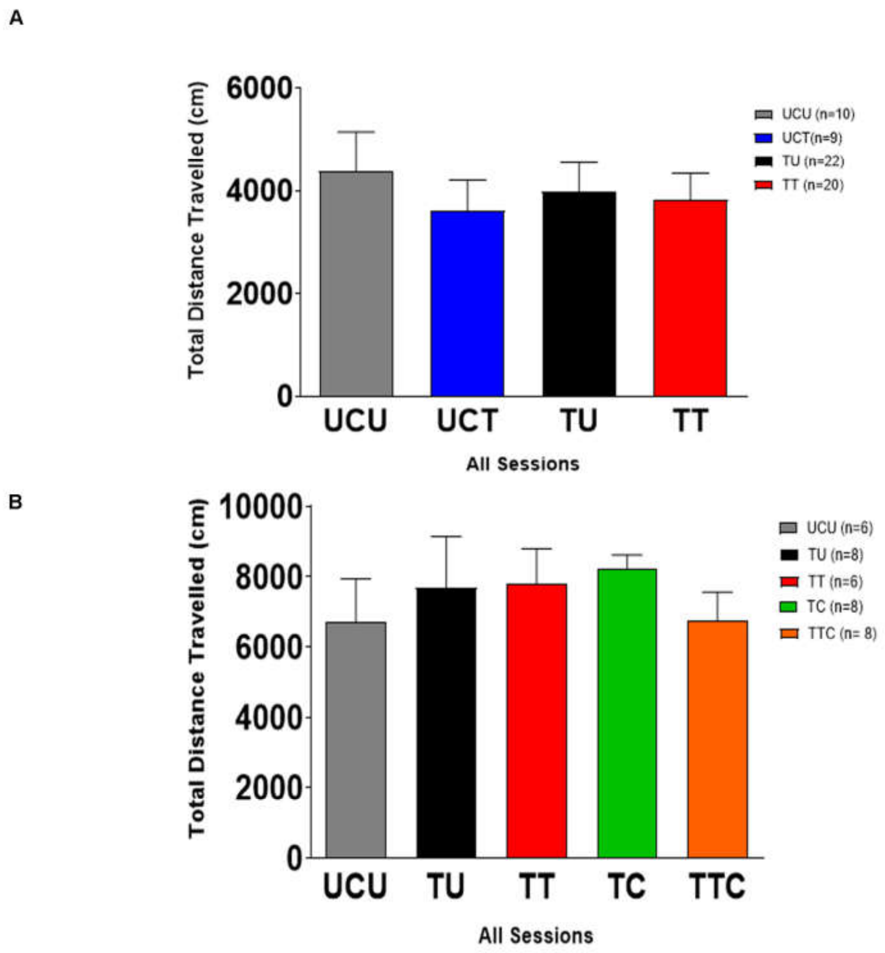

Figure S2. (**A**,**B**) Total distance travelled by Open Field Test. In all sessions, no significant difference in the total distance travelled by the different group of rats in the whole OFT. One-way repeated measures ANOVA with post hoc Fisher (LSD) was used to validate significance. *, **, ***, **** indicate p values ≤0.05; 0.01 and 0.001, 0.0001, respectively. p-values less than 0.05 were considered significant. SEM ± are reported. For both panels, ordinary one-way repeated measures ANOVA with post hoc Fisher least (LSD) was used to validate significance. For (**A**), UCU, n = 10; UCT, n = 9; TU, n = 22; TT, n = 20. For (**B**), UCU, n = 6; TU, n = 8; TT, n = 6; TC, n = 8, TTC, n = 8.
